# Prediction of individualised 6-month mortality risk in opioid use disorder

**DOI:** 10.1192/bjp.2025.10313

**Published:** 2025-07-07

**Authors:** Emmert Roberts, John Strang, Eve Taylor, Jamie Crummy, Tim Lowden, Chioma Amasiatu, Brian Eastwood

**Affiliations:** National Addiction Centre and the Department of Psychological Medicine, Institute of Psychiatry, Psychology and Neuroscience, Kings College London, the South London and the Maudsley NHS Foundation Trust and the Office for Health Improvement and Disparities, Department of Health and Social Care; National Addiction Centre, Institute of Psychiatry, Psychology and Neuroscience, Kings College London and the South London and the Maudsley NHS Foundation Trust; National Addiction Centre, Institute of Psychiatry, Psychology and Neuroscience, Kings College London; Office for Health Improvement and Disparities, Department of Health and Social Care

**Keywords:** Mortality, Risk Prediction, Opioid Use Disorder, Death, Prognosis, Epidemiology

## Abstract

**Background:**

People with opioid use disorder (OUD) have substantially higher standardised mortality rates compared to the general population. However, lack of individualised prognostic information presents challenges to personalisation of addiction treatment delivery.

**Aims:**

We developed and validated the first prognostic models to estimate 6-month all-cause and drug-related mortality risk for people diagnosed with OUD using indicators recorded at baseline assessment in addiction services in England.

**Method:**

Thirteen candidate prognostic variables, including sociodemographic, injecting status, health and mental health factors, were identified from nationally linked addiction treatment, hospitalisation and death records from 1^st^ April 2013 - 1^st^ April 2022. Multivariable Cox regression models were developed with a fractional polynomial approach for continuous variables, and missing data addressed using multiple imputation by chained equations (MICE). Validation was undertaken using bootstrapping methods. Discrimination was assessed using Harrel’s C and D-statistics alongside examination of observed to predicted event rates and calibration curve slopes.

**Results:**

Data were available for 236,064 people with OUD with 2,427 deaths due to any cause including 1,289 due to drug-related causes. Both final models demonstrated good optimism-adjusted discrimination and calibration, all-cause and drug-related models respectively demonstrating Harrell’s C statistics of 0.73 (95% Confidence Interval (CI) 0.71-0.75) and 0.74 (95% CI 0.72-0.76), D-statistics of 1.01 (95% CI 0.95-1.08) and 1.07 (95% CI 0.98-1.16) and calibration slopes of 1.01 (95% CI 0.95-1.08) and 1.01 (95% CI 0.94-1.10).

**Conclusions:**

We developed and internally validated Roberts’ OUD (ROUD) Mortality Risk, the first models to accurately quantify individualised absolute 6-month mortality risks in people with OUD presenting to addiction services. Independent validation is warranted to ensure these models have optimum utility to assist wider future policy, commissioning and clinical decision-making.

## Introduction

In 2022 England reported its highest number of drug-related deaths on record. (1) Opioids were implicated in almost half of all drug-related deaths whilst opioid use disorder (OUD) was a problem for half of all adults accessing community addiction services. (2) Over the past eight years, between one and two percent of all adults accessing community addiction services with OUD have died each year whilst receiving treatment. (2) Professionals working in community addiction services play a key role in both the delivery of evidenced-based treatment and in the provision of prognostic information to individuals with OUD. However, despite good understanding that, on average, people with OUD have up to ten times higher standardised mortality rates compared with the general population, (3, 4) uncertainty regarding individual prognosis and mortality risk presents challenges to addiction services in providing individuals with accurate personalised risk information, prioritisation of finite resources and appropriate targeting of interventions.

Expansion in the use of clinical informatics and precision medicine has revolutionised the care provided in many healthcare sectors, (5) however development and validation of prognostic risk models in populations of people with OUD has been relatively limited. This is despite multiple systematic reviews examining individual prognostic risk-factors for mortality among people with OUD, (3, 4, 6–8) and a number of studies recently developing models in populations routinely prescribed opioids (e.g., to examine the risk of developing OUD or the risk of opioid-overdose). (9, 10) To our knowledge, no models have been developed examining mortality risk in people with a diagnosis of OUD presenting to community addiction services. Models that examine both all-cause and drug-related mortality risks could provide useful information and assistance to both individuals and professionals to make collaborative treatment decisions at the clinically important point of entering addiction treatment.

Potential explanations for the relative paucity of prognostic modelling studies in this area include the required sample size and number of events, and lack of centralised data repositories which include linked accurate prognostic and outcome information from healthcare and administrative agencies. England is unusual, having recently established and validated a ten-year national data linkage between all individuals presenting to community addiction services and their hospitalisation and death records (n>900,000). (11) This is coupled with the fact that all people in England, regardless of overseas visitor or immigration status, are able to access community addiction services free of charge at the point of delivery and hence there is a relative absence of a privately funded treatment system. (12) The availability and coverage of this national linked dataset thus provides a rare opportunity to develop and validate adequately powered prognostic models within this population.

### Objectives

This study aimed to develop and validate two models, one to estimate 6-month all-cause mortality risk and one to estimate 6-month drug-related mortality risk for people with OUD from prognostic indicators routinely recorded during initial assessment at community addiction services in England.

## Methods

The complete protocol has been previously published, (13) with the study designed and reported in accordance with the Transparent Reporting of multivariable prediction models for Individual Prognosis Or Diagnosis that use regression or machine learning methods, the TRIPOD + AI statement. (14) The completed TRIPOD + AI checklist can be found in the online supplementary material as table S1. The work benefitted throughout from input from the South London and the Maudsley Biomedical Research Centre Data Linkage Service User and Carer Advisory Group which includes experts with lived experience of OUD. (15)

### Setting

The study utilises a national English dataset which contains linked individual records from three sources: 1) The National Drug Treatment Monitoring System (NDTMS) - a centralised database, collated and maintained by the Department of Health and Social Care (DHSC), which receives monthly input from all adult statutory community addiction services in England. (16) NDTMS contains individual-level data on an individual’s sociodemographic characteristics (date of birth, sex, housing status etc.), what substances the individual is using problematically, any interventions received, and measures of treatment success. 2) Hospital Episode Statistics (HES) - a centralised database, collated and maintained by the National Health Service (NHS), which collects all information pertaining to NHS inpatient hospitalisation in England. (17) HES covers all NHS inpatient admissions, including any admission to private or third sector hospitals subsequently reimbursed by the NHS, and is estimated to contain >99% of all inpatient hospital activity in England. An inpatient hospital admission includes any secondary care-based activity requiring a hospital bed, thus includes day cases, and both planned and emergency admissions, in physical and mental health settings. HES does not cover accident and emergency (A&E, emergency department) attendances, nor outpatient bookings, these data being held in separate databases. 3) Office of National Statistics (ONS) - a centralised database that contains official death certification records for those individuals that have died. The overall structure of the linked NDTMS-HES-ONS data is clustered with individuals attending one of 150 uniquely commissioned statutory community addiction services across each local authority area in England.

Approval to conduct the linkage analyses was granted under regulation 3 of the Health Service (Control of Patient Information) Regulations 2002, following review by the Caldicott Advisory Panel (CAP) (Ref: CAP-2019-06) and the Department of Health and Social Care Office of Data Protection (ODP). NDTMS data were available from 1^st^ April 2013 - 1^st^ April 2022, containing data on n=236,064 unique individuals, aged 18 or over, who attended community addiction services for treatment of problematic opioid use at least once within that timeframe. A standardised clinical history supported with urine drug screen investigation are used within community addiction services to confirm and record a diagnosis of OUD. Linked HES and ONS data are available for individuals detailing any subsequent death records and any individual hospital admissions since HES database inception in 1997. (11) The linked database can only be accessed by DHSC staff working on the project with all records stored for a minimum of 5 years after study completion. The studied timeframe deviates from the published protocol, the end date of the study window previously specified as 1^st^ April 2023. This deviation was necessary because accurate death record outcome data was not yet available for all individuals presenting to addiction services after 1^st^ April 2022.

### Candidate predictor variables

The prognostic indicators for consideration in the multivariable model were initially identified from a systematic search of review articles and their underlying included studies which examined demographic and clinical features associated with increased mortality for people with OUD. (3, 4, 6–8, 18) All candidate predictor variables that were significantly associated with all-cause mortality were extracted and, those which were available within the linked NDTMS-HES-ONS records, discussed with patients and clinicians over a series of three consensus meetings. Given the aim was to create a model that could be readily incorporated into routine clinical care, within time-pressured services, a parsimonious approach was taken to a selection of prognostic indicators with clinician and patient involvement suggesting that, ideally, no more than ten variables should be included in a final model. An agreed consensus set of prognostic indicator variables was subsequently extracted from NDTMS-HES-ONS records retrospectively from the time of baseline assessment (i.e., the point at which the patient initially presents for treatment) at the community addiction service, designated as time zero (*t*0). Following discussion, the protocol initially identified twelve candidate prognostic indicator variables; however, following additional patient and clinician input during model development, one supplementary candidate variable, the binary of whether a person had ever previously been in addiction treatment, was added as a candidate predictor. Descriptions and structure of each candidate variable can be found in table one. (19, 20)

### Outcome Measures

The binary outcomes of all-cause and drug-related mortality were assessed prospectively for each individual at any point up to 6 months after *t*0. This timepoint was chosen following clinician and patient feedback, as it was thought to reflect a time-horizon of sufficient duration to potentially encourage risk factor modification. Drug-related death follows the definition used by the ONS when reporting official national statistics for deaths related to drug-poisoning. The death certificate International Classification of Diseases, Tenth Revision (ICD-10) codes for drug-related death can be found in table S2 in the online supplementary material and include deaths due to mental and behavioural disorders due to drug use (ICD-10: F11-F16, F18-F19), assault (ICD-10: X85) and poisoning of accidental, intentional and undetermined intent (ICD-10: X40-X44, X60-X64 and Y10-Y14).

### Sample Size

The minimum required sample size for time-to-event model development is based on estimated event rates of the prediction models outcomes. (21) Given that the drug-related death event rate is by definition smaller than the all-cause death rate, and thus requires a larger sample size, this outcome was chosen for sample size calculation. Estimation used the ‘*pmsampsize’* command and in the absence of any reported Cox-Snell R-squared values from previously developed models, we aimed to develop a model with a minimal anticipated Harrel’s C statistic (a measure of discrimination similar to the area under a Receiver Operating Characteristic (ROC) curve but taking account of the censored nature of the data) of 0.70, allowing a maximum shrinkage of 10% to minimize potential overfitting. (22) A maximum total of 12 candidate predictors was originally planned with an estimated event rate based on a previous cohort study which reported 0.0134 drug-related deaths per person year. (18) This estimated a minimum required sample size was 2487 participants and 51 events.

### Missing data

Complete outcome data was available for all individuals with complete candidate predictor information available for eight variables. Of the five candidate predictors with missing data (injecting status, HIV positivity, hepatitis C RNA positivity, prison referral and accommodation need) the fraction of missing information (FMI) and its assumed missingness mechanism was assessed for each variable with all deemed to at least reasonably fulfil the missing at random (MAR) assumption (i.e., the probability of a value’s being missing in one variable was not deemed related to the probability of missing data in another variable). Missing data was addressed using multiple imputation by chained equations (MICE), the number of imputations set at *m*=50 based on the highest FMI. (23) Rubin’s rules were used to combine the results across imputed datasets. (24)

### Statistical analysis

Multivariable Cox regression models were developed using backwards elimination with the level of alpha for variable exclusion set at 0.157, as recommended based on the Akaike Information Criterion (AIC). (25, 26) Nonlinearity of continuous variables was addressed by using a multivariable fractional polynomial approach, an established technique for transforming non-linear continuous variables when developing a backwards elimination model. (27) Model discrimination was assessed through the calculation of the Harrel’s C and D-statistic (a measure of discrimination where higher values indicate better discrimination) with calibration curve slopes and the ratio of the observed to predicted event rates examined. (27, 28) Validation was undertaken using bootstrapping resampling methods, which account for bias due to over-fitting more accurately than split sample cross-validation approaches, with the model development process repeated in 1000 bootstrap samples to allow calculation of optimism-adjusted discrimination and calibration measures. (29) Performance was also evaluated by calculation of Harrell’s C statistics for each cluster (i.e., each of the 150 individual statutory community addiction services) and the results combined using random effects meta-analysis, weighted by the number of events per service. Between-cluster heterogeneity was assessed using the I^2^ statistic. (28). All analyses were conducted in Stata version 18.0 (StataCorp, College Station, Texas, USA).

## Results

Data were available for 236,064 people with OUD. There were 2,427 deaths due to any cause and 1,289 deaths due to a drug-related cause within six months of individuals most recent presentation to community addiction services in England. Baseline characteristics of the whole sample and those dying due to any or drug-related causes within six months are available in table two.

### Development

Table three shows the optimism-adjusted hazard ratios (aHRs) for both final models. In both models, the final model and all variables met the assumption of proportional hazards.

#### All-cause mortality

All variables were preserved in the all-cause mortality model except HIV positivity and polysubstance use, leading to a final model event per variable (EPV) rate of 2,427/13=186.69. Figure S1 in the online supplementary material shows the graphical representation of the aHRs for the fractional polynomial terms for age, this was treated as a cubic function in the final model. Of the variables included in the final model optimism - adjusted increased individual risk of all-cause mortality ranged between a 13% increase (95%CI, 2 - 26%) for having a non-urgent housing problem to a 123% increase (95%CI, 34 - 271%) for having a previous involuntary mental health admission.

#### Drug-related mortality

All variables were preserved in the drug-related mortality model except HIV positivity, polysubstance use and age, neither fractional polynomial terms at the first or second degree reaching the prespecified significance for inclusion. This led to a final model EPV rate of 1,289/12=107.42. Of the variables included in the final model optimism-adjusted increased individual risk of drug-related mortality ranged between a 12% increase (95%CI: 0 - 27%) increase for having a previous acute hospital admission to a 187% (95%CI, 144 - 238%) increase for being a person who currently injects.

Table S3 in the online supplementary material shows the complete case analysis (i.e., the results based on people with only complete candidate predictor variable data), the aHRs showed broadly similar trends in both final models using the multiply imputed data.

### Validation

Table four shows the optimism-adjusted performance statistics for both final models.

#### Discrimination

##### All-cause mortality

The optimism-adjusted final model explained 20% of the variation in time to all-cause mortality (R^2^_D_), the D statistic was 1.01, and the Harrell’s C statistic was 0.73. Figure S2 in the online supplementary material shows the forest plot of Harrel’s C statistics across individual community addiction services in England, services with fewer deaths had wider variation in the Harrel’s C statistic than services with more events. Four services had no deaths over the studied timeframe and were not included in the meta-analysis. The summary pooled Harrel’s C statistic was 0.77 (95%CI, 0.75 - 0.79) ranging from 0.66 (95%CI, 0.57 - 0.74) to 0.99 (95%CI, 0.98 - 1.00). The I^2^ value (i.e., the percentage of total variation in Harrel’s C statistics explained by between service heterogeneity) was 99.5% (95%CI, 96.0% - 99.8%).

##### Drug-related mortality

The optimism-adjusted final model explained 21% of the variation in time to drug-related mortality (R^2^_D_), the D statistic was 1.07, and the Harrell’s C statistic was 0.74. Figure S3 in the online supplementary material shows the forest plot of Harrel’s C statistics. Six services had no drug-related deaths over the studied timeframe and were not included in the meta-analysis. The summary pooled Harrel’s C statistic was 0.81 (95%CI, 0.80 - 0.82) ranging from 0.63 (95%CI, 0.47 - 0.79) to 0.99 (95%CI, 0.99 - 1.00). The I^2^ value was 99.9% (95%CI, 99.7% - 100.0%).

#### Calibration

##### All-cause mortality

The optimism-adjusted calibration slope was 1.01 (0.95 - 1.08). The observed all-cause mortality risk at six months was 1.03% (0.99% - 1.07%) compared to a mean individual predicted risk of 5.02% (5.00% - 5.04%).

##### Drug-related mortality

The optimism-adjusted calibration slope was 1.01 (0.94 - 1.10). The observed drug-related mortality risk at six months was 0.55% (0.52% - 0.58%) compared to a mean individual predicted risk of 2.19% (2.18% - 2.20%).

Table five depicts clinical examples of six-month all-cause and drug-related mortality risk for individuals with OUD presenting to community addiction services in England with a web calculator for individualised risks available at https://connect.calcapp.net/?app=4pekem.

## Discussion

We developed and internally validated Roberts’ OUD (ROUD) Mortality Risk, two multivariable prognostic models to estimate 6-month all-cause and drug-related mortality risk for people with OUD presenting for baseline assessment at community addiction services in England. To our knowledge no previous models have been developed examining these outcomes in the studied population, which may provide clinically useful information and assistance to both patients and professionals when making treatment and care decisions.

Both models performed well in terms of discriminatory ability, with optimism-adjusted Harrell’s C above 0.7 and D-statistics above 1.0. Only around one fifth of the variation in time to death was explained by each model. Whilst addition of further predictors has the potential to increase this statistic, we were mindful throughout model development and consultation with clinicians and patients, of the need for balance between model parsimony and performance. Models with an excessive number of parameters, or which use parameters that may require complex questioning or interpretation, are likely to face implementation problems and thus lack clinical utility within time-pressured services, particularly if risk scoring is completed by professionals with a range of levels of experience and training. (30) As there was substantial heterogeneity in discriminative ability between services across England, the pooled Harrel’s C statistics should be interpreted with caution due to the large number of services with a low EPV rate, the pooled estimate likely overestimating discriminative performance. However, it was reassuring that all individual service Harrel’s C estimates remained >0.65 for both models. Patient and clinician consultation prior to model development suggested that a ten variable model would be optimal. Both final models contain eleven variables or fewer and are thus roughly in accord with this consensus. (30) Mean predicted mortality risks were higher in both models than the observed risk, this likely being indicative of the risk reduction associated with being engaged in addiction treatment, and in particular for individuals receiving opiate agonist therapy (OAT). Indeed, recent estimates demonstrate a three and a half times higher drug-related death rate for those not in receipt of OAT compared with those on OAT after adjustment for confounders, (18) and in a fully adjusted post-hoc model, which we developed to assess the impact of the binary variable of if individuals had remained engaged in addiction treatment at six-months from baseline assessment, demonstrated a significantly decreased risk of both all-cause and drug-related mortality at six-months if individuals remained in treatment. As these models are intended to calculate risk at baseline assessment at community addiction services, a point at which, by definition, individuals are currently not in receipt of any treatment, including OAT, they allow individualised risk calculation at a clinically important point of time and allow individuals and professionals to contemplate risks without subsequent treatment. Individualised risk information provision at this juncture may thus have the potential to both result in internally generated behaviour change to both address modifiable risk factors within individuals with OUD and remain engaged in treatment, (31) and also to assist professionals in the prioritisation of finite resources, such as prescriber availability, and ensure that individuals with elevated risks are actively supported to remain in treatment services. (32) There may be concern that providing individualised mortality risk could result in emotional distress for both patients and professionals, however patient and clinician involvement suggested this information would be welcomed, compared to the current clinical reality of stating that there was increased risk but with little personalised quantification. Indeed, studies in other settings have demonstrated perceived utility of provision of this type of individualised health information. (33) It is notable that no fractional polynomial of any degree resulted in the inclusion of age in the drug-related mortality model, this is in accordance with previous research that challenges the “ageing cohort” theory of increase in drug-related deaths which do not appear to be driven by age. (34)

There are multiple strengths to the study including the comprehensive, non-selective and national nature of the dataset and the substantial involvement of clinicians and patients from the outset to through model development, validation and interpretation. The protocol pre-publication, alongside documentation of any deviation, and study reporting in accordance with the TRIPOD + AI statement also are notable strengths. (35) There remain however several potential limitations. All prognostic indicator variables were collected retrospectively from an administrative dataset, the underlying data for which has been supplied by addiction treatment, hospital and death registration services. There is therefore potential information bias and risk of lack of availability of some predictor variables, as noted particularly with HIV and Hepatitis C, if submitted documentation is incomplete. While relying on routinely documented clinical information as the source of prognostic information has limitations, this approach has been utilised frequently and does reflect how the model would be used in clinical practice, with some information potentially not being available to professionals or patients at the time of baseline assessment. (36) The model will benefit from independent validation in other samples and subsequent examination of its utility in clinical practice and acceptability among professional and patient groups with potentially suitable datasets identified in both Wales and Australia. (37, 38) Given this, we have ensured reporting of model baseline survivor and linear predictor values. Continued co-production through independent validation and implementation with clinicians and patients will remain a key requirement.

Whilst there have been significant expansions and understanding in the use of machine learning methods to develop prognostic models across healthcare sectors, initial patient and clinician feedback demonstrated reticence to employ these within the context of mortality prediction in OUD. The perception of a ‘black box’ or lack of transparent understanding of what prediction outcome scores were based on, and the relative infancy of clinical informatics within the OUD field led to concerns about clinical utility, and implementation within community addiction services. (39) Clinicians were comfortable with clinical risk tools developed using classical statistical methods, and their corollaries used in other areas of healthcare, (36) and welcomed their potential expansion within addiction settings. However, there was concern among patients that results from machine learning methods would not be believed, and explanation of algorithms could create difficulties in conveying the predictive information to individuals accessing services. As such traditional statistical methods were chosen to develop this model.

## Conclusions

Standardised all-cause and drug-related mortality rates are significantly elevated among people with OUD, and despite a significant body of literature describing individual prognostic risk factors, often clinical judgment alone is used to consider individual prognosis and the prioritisation of treatment interventions in addiction treatment services. Whilst other areas of medicine routinely incorporate risk tools into care to assist clinical decision making, (36, 40) clinical informatics within the addiction field has been somewhat slower to progress. Given the significant elevated mortality risks within this population the development and validation of the first individualised prediction models that demonstrate good optimism-adjusted discrimination and calibration appears timely, warranted and urgent. This is the first stage of model assessment. Independent validation and demonstration of clinical utility of both models are necessary next steps to ensure buy in from professionals, policymakers and patients if they are to be valued and successfully implemented.

## Supplementary Material

Supplementary Material

## Figures and Tables

**Table one:** Candidate predictor variables

Candidate Predictor Variable	Variable structure in NDTMS-HES-ONS
Age	Continuous
Sex	Binary: 0: Female 1: Male
History of injecting behavior	Categorical: 0: Never injected 1. Previously injected (but not currently) 2. Currently injecting
HIV Positivity^1^	Binary: 0: No 1: Yes
Hepatitis C RNA Positivity^1^	Binary: 0: Negative (never infected or cleared by treatment) 1: Positive
Polysubstance Use: Number of substances used problematically^2^	Categorical: 0: One problematic substance 1: Two problematic substances 2: ≥3 problematic substances
Problematic alcohol use^2^	Binary: 0: No problematic use of alcohol 1: Problematic use of alcohol
Problematic benzodiazepine use^2^	Binary: 0. No problematic use of any benzodiazepine 1. Problematic use of any benzodiazepine
Accommodation Need	Categorical: 0: No housing problem - Owner occupier - Tenant – private landlord/housing association/Local - Authority/ registered landlord/ arm’s length management organisation - Approved premises - Supported housing/hostel - Traveler - Own property - Settled mainstream housing with friends/family - Shared ownership scheme 1: Housing problem i.e., - Staying with friends/family as a short-term guest - Night winter shelter - Direct Access short stay hostel - Short term B & B or other hotel - Placed in temporary accommodation by Local Authority - Squatting 2: No Fixed Abode - Urgent Housing Problem i.e., - Lives on streets/rough sleeper - Uses night shelter (night-by-night basis) /emergency hostels - Sofa surfing/sleeps on different friend’s floor each night
Prison referral	Binary: 0. Referred to the drug service by any source other than prison 1. Referred to the drug service from prison
Acute inpatient hospital admission^3^	Binary: 0. No acute inpatient hospital admissions within the person’s lifetime 1. Inpatient acute hospital admission within the person’s lifetime
Mental health inpatient hospital admission^3^	Binary: 0. No inpatient involuntary mental health hospital detention within the person’s lifetime 1. Inpatient involuntary mental health hospital detention within the person’s lifetime
Previous history of addiction treatment^4^	Binary: 0. The person has never previously had an episode of addiction treatment 1. The persons has previously had an episode of addiction treatment

NDTMS-HES-ONS (National Drug Treatment Monitoring System - Hospital Episode Statistics - Office of National Statistics)

1 Available from 2020 onwards;

2 Problematic use as deemed by the assessing clinician;

3 Variation from protocol which specified that the acute and mental health inpatient hospital admissions be within the last six months, following continued patient and clinician input during model development, this was revised to lifetime admission to hospital which was thought to be an easier question to ask individuals with OUD and the strict timeframe could lead to implementation issues with checking records;

4 Variation from protocol this predictor was added following patient and clinician input during model development.

**Table two:** Baseline characteristics of people with opioid use disorder presenting to community addiction services in England between 1^st^ April 2013 and 1st April 2022.

		Full sample n	Died due to any cause within six months n	Died due to a drug-related cause within six months n
All		236,064 (100%)	2,427 (100%)	1,289 (100%)
Mean (SD) age (years)		43.4 (9.2)	46.2 (9.6)	43.8 (8.6)
Sex	Female	63,443 (26.9%)	538 (22.2%)	291 (22.6%)
	Male	172,621 (73.1%)	1,889 (77.8%)	998 (77.4%)
History of injecting behavior	Never injected	98,542 (41.7%)	565 (23.3%)	229 (17.8%)
	Previously injected (but not currently)	72,085 (30.5%)	944 (38.9%)	517 (40.1%)
	Currently injecting	63,542 (26.9%)	892 (36.8%)	532 (41.3%)
	Missing^1^	1,895 (0.8%)	26 (1.1%)	11 (0.9%)
HIV Positivity	Negative	97,912 (41.5%)	271 (11.2%)	140 (10.9%)
	Positive	2,132 (0.9%)	11 (0.5%)	8 (0.6%)
	Missing^1^	136,020 (57.6%)	2,145 (88.4%)	1,141 (88.5%)
Hepatitis C RNA Positivity	Negative	86,931 (36.8%)	417 (17.2%)	233 (18.1%)
	Positive	10,443 (4.4%)	148 (6.1%)	79 (6.1%)
	Missing^1^	138,690 (58.8%)	1,862 (76.7%)	977 (75.8%)
Polysubstance Use: Number of substances used problematically	1	63,000 (26.7%)	565 (23.3%)	277 (21.5%)
	2	99,822 (42.3%)	997 (41.1%)	505 (39.2%)
	≥3	73,242 (31.0%)	865 (35.6%))	507 (39.3%)
Problematic alcohol use	No	203,307 (86.1%)	1,875 (77.3%)	1014 (78.7%)
	Yes	32,757 (13.9%)	552 (22.7%)	275 (21.3%)
Problematic benzodiazepine use	No	219,926 (93.2%)	2,189 (90.2%)	1113 (86.3%)
	Yes	16,138 (6.8%)	238 (9.8%)	176 (13.7%)
Accommodation Need	No housing problem	153,346 (64.7%)	1,595 (65.7%)	829 (64.3%)
	Housing problem	31,677 (13.4%)	404 (16.7%)	221 (17.2%)
	Urgent housing problem - NFA	25,370 (10.8%)	364 (15.0%)	217 (16.8%)
	Missing^1^	25,671 (10.9%)	64 (2.6%)	22 (1.7%)
Prison referral	Not referred from prison	203,012 (86.0%)	1,983 (81.7%)	1,012 (78.5%)
	Referred from prison	31,774 (13.5%)	427 (17.6%)	268 (20.8%)
	Missing^1^	1,278 (0.5%)	17 (0.7%)	9 (0.7%)
Acute inpatient hospital admission	None	76,942 (32.6%)	652 (26.9%)	375 (29.1%)
	Previous admission	159,122 (67.4%)	1,775 (73.1%)	914 (70.9%)
Mental health inpatient hospital admission	None	235,499 (99.8%)	2,412 (99.4%)	1,280 (99.3%)
	Previous admission	565 (0.2%)	15 (0.6%)	9 (0.7%)
Previous history of addiction treatment	First treatment episode	83,222 (35.3%)	469 (19.3%)	248 (19.2%)
	Previous treatment episode	152,842 (64.7%)	1,958 (80.7%)	1,041 (80.8%)

SD Standard Deviation; NFA No Fixed Abode;

1 Proportion of missing values imputed as variable values can be found in the online supplementary material in table S4 in the online supplementary material

**Table three:** Optimism-adjusted hazard ratios (95% confidence interval) for six month all-cause and drug-related mortality in individuals with opioid use disorder presenting to community addiction services in England.

		All-cause mortality	Drug-related mortality
Age^1^		1.00 (1.00 - 1.00)	N/A
Sex	Female	Reference	Reference
	Male	1.15 (1.04 - 1.26)	1.16 (1.01 - 1.32)
History of injecting behavior	Never injected	Reference	Reference
	Previously injected (but not currently)	1.90 (1.71 - 2.12)	2.57 (2.19 - 3.02)
	Currently injecting	2.06 (1.84 - 2.30)	2.87 (2.44 - 3.38)
Hepatitis C RNA Positivity	Negative	Reference	Reference
	Positive	1.29 (1.12 - 1.49)	1.26 (1.03 - 1.53)
Problematic alcohol use	No	Reference	Reference
	Yes	1.73 (1.57 - 1.91)	1.67 (1.46 - 1.91)
Problematic benzodiazepine use	No	Reference	Reference
	Yes	1.34 (1.17 - 1.53)	1.86 (1.58 - 2.18)
Accommodation need	No housing problem	Reference	Reference
	Housing problem	1.13 (1.02 - 1.26)	1.12 (0.96 - 1.30)
	Urgent housing problem - NFA	1.17 (1.04 - 1.32)	1.20 (1.03 - 1.41)
Prison referral	Not referred from prison	Reference	Reference
	Referred from prison	1.35 (1.21 - 1.50)	1.46 (1.27 - 1.68)
Acute inpatient hospital admission	None	Reference	Reference
	Previous admission	1.18 (1.08 - 1.30)	1.13 (1.00 - 1.27)
Mental health inpatient hospital admission	None	Reference	Reference
	Previous admission	2.23 (1.34 - 3.71)	2.67 (1.39 - 5.16)
Previous history of addiction treatment	First treatment episode	Reference	Reference
	Previous treatment episode	1.93 (1.74 - 2.12)	1.89 (1.64 - 2.18)
Baseline survivor function^2^		0.9917	0.9958
Mean linear predictor (SD, range)		1.62 (0.66, 0.03 - 5.17)	1.40 (0.73, 0.00 - 4.30)

1 Modelled as a cubic function in all-cause mortality model

2 Continuous covariates set at their means and categorical or binary variables set at their reference values

**Table four:** Mean (95% confidence interval) performance of six month all-cause and drug-related mortality prediction models in people with opioid use disorder presenting to community addiction services in England

	All-Cause Mortality		Drug-Related Mortality	
	Original Apparent	Optimism Adjusted	Original Apparent	Optimism Adjusted
Harrell’s C statistic	0.73 (0.71 - 0.75)	0.73 (0.71 - 0.75)	0.74 (0.72 - 0.76)	0.74 (0.72 - 0.76)
D Statistic	1.02 (0.96 - 1.07)	1.01 (0.95 - 1.08)	1.09 (1.00 - 1.17)	1.07 (0.98 - 1.16)
R^2^_D_	0.20 (0.18 - 0.22)	0.20 (0.18 - 0.22)	0.22 (0.19 - 0.25)	0.21 (0.19 - 0.24)
Calibration slope	1 (0.94 - 1.06)	1.01 (0.95 - 1.08)	1 (0.92 - 1.08)	1.01 (0.94 - 1.10)

**Table five:** Clinical examples of all-cause and drug-related six-month mortality risk for people with opioid use disorder presenting to community addiction services in England.

	Examples			
	1	2	3	4
Age	18	52	66	53
Sex	Female	Male	Male	Male
History of injecting behavior	Never injected	Previously injected	Currently injecting	Currently injecting
Hepatitis C RNA Positivity	Negative	Negative	Negative	Negative
Problematic alcohol use	No	Yes	Yes	Yes
Problematic benzodiazepine use	No	No	No	Yes
Accommodation need	No housing problem	No housing problem	Housing problem	Housing Problem
Prison referral	No	No	No	No
Acute inpatient hospital admission	No	No	Yes	Yes
Mental health inpatient hospital admission	No	No	No	Yes
Previous history of addiction treatment	No	Yes	Yes	Yes
All-cause mortality predicted risk (%)	0.86%	10.32%	25.22%	38.63%
Drug-related mortality predicted risk (%)	0.42%	3.90%	5.44%	24.26%
