# Supplementary material for: Prediction of individualised 6-month mortality risk in opioid use disorder
Source: Br J Psychiatry. Author manuscript; Available in PMC 2025 Jul 28. (PMC7617949; doi:10.1192/bjp.2025.10313)
Supplement: Supplementary Material [file EMS205653-supplement-Supplementary_Material.pdf]

Online Supplementary Material

Contents

Figures

S1. Graphical representation of the adjusted Hazard Ratios (aHRs) for the fractional polynomial terms for age in the all-cause mortality model

S2. Forrest plot of discrimination performance (Harrell's C statistic) for the all-cause mortality at six-months model across 146 community addictions services in England

S3. Forrest plot of discrimination performance (Harrell's C statistic) for the drug-related mortality at six-months model across 144 community addictions services in England

Tables

S1. TRansparent reporting of a multivariable prediction model for Individual Prognosis Or Diagnosis (TRIPOD+AI) checklist for the reporting of prediction model studies (1)

S2: International Classification of Diseases, Tenth Revision (ICD-10) codes used to define drug-related deaths

S3. Adjusted hazard ratios (95% confidence interval) for the complete case analysis of six month all-cause and drug-related mortality in individuals with opioid use disorder presenting to community addiction services in England.

S4. Proportion of missing values imputed as variable values following Multiple Imputation by Chained Equations (MICE)

References

Figures

Figure S1. Graphical representation of the adjusted Hazard Ratios (aHRs) for the fractional polynomial terms for age in the all-cause mortality model

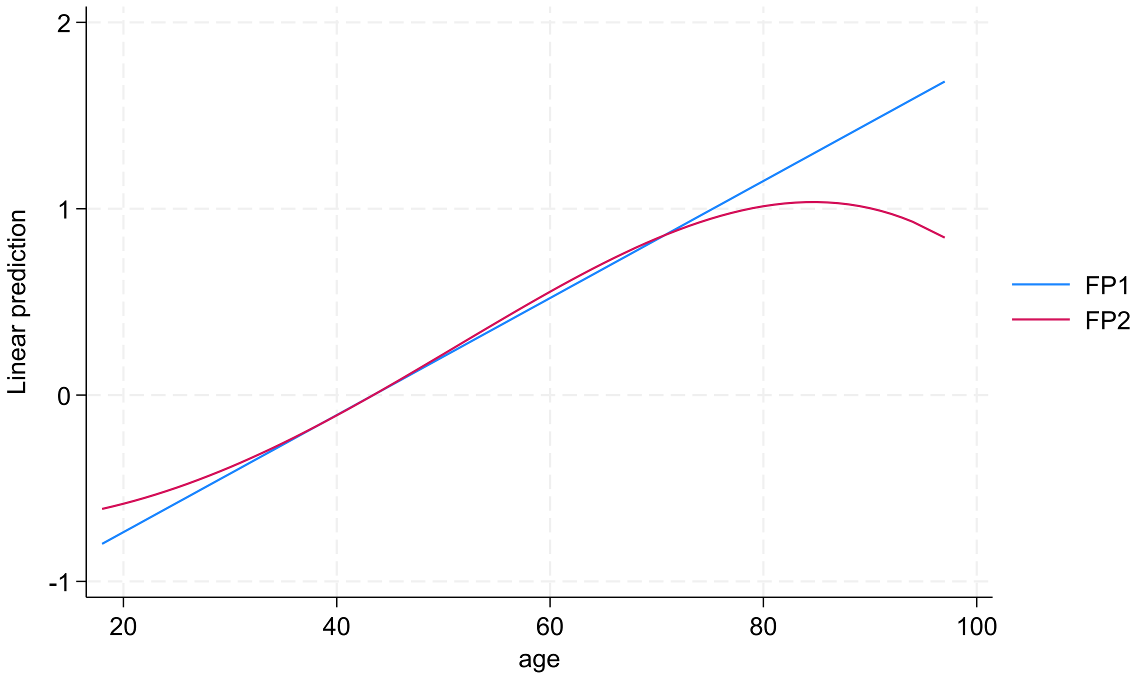

FP = Fractional Polynomial at the first (FP1) or second (FP2) degree

Figure S2: Forrest plot of discrimination performance (Harrell's C statistic) for the all-cause mortality at six-months model across 146 community addictions services in England

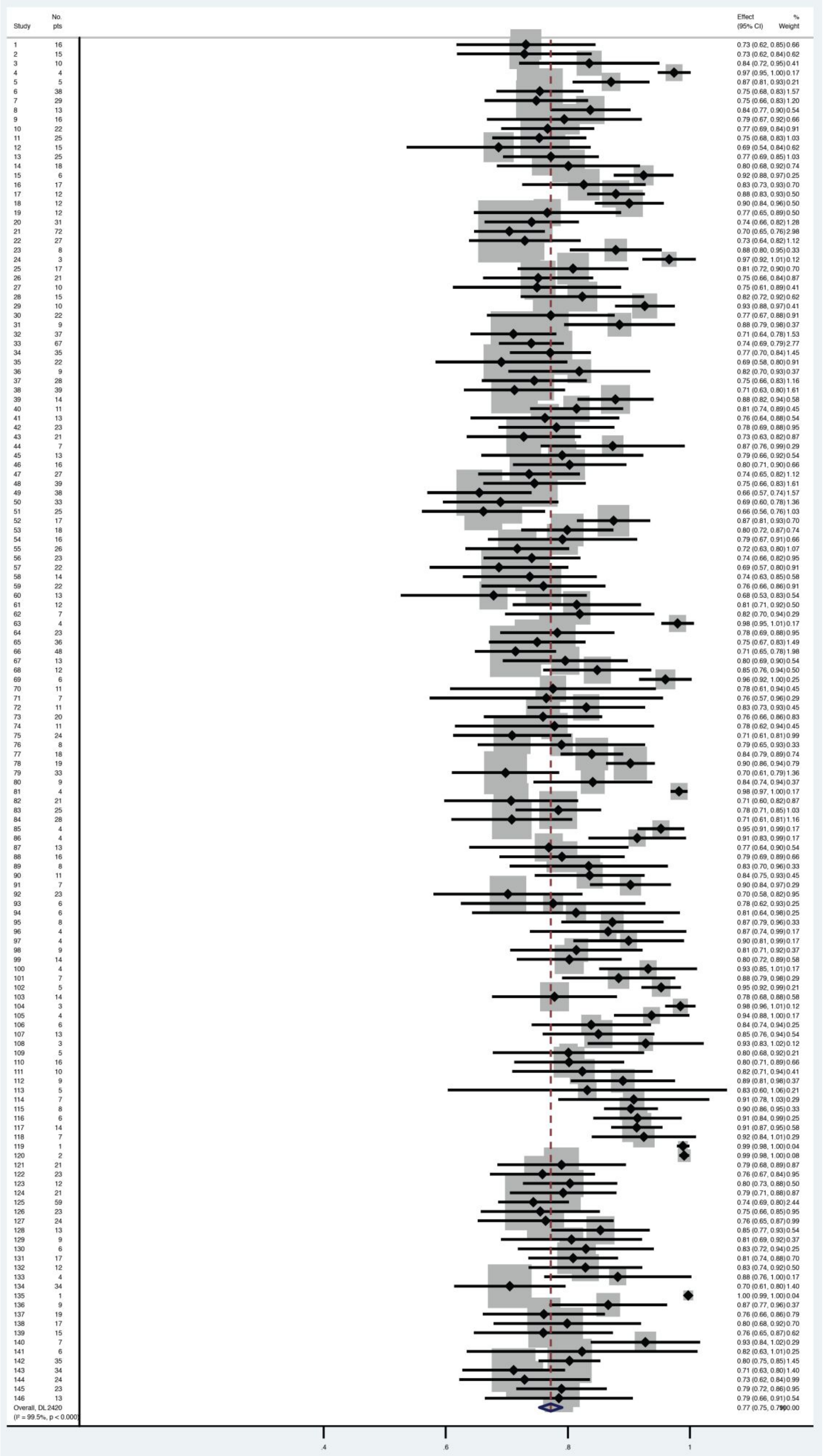

S3. Forrest plot of discrimination performance (Harrell's C statistic) for the drug-related mortality at six-months model across 144 community addictions services in England

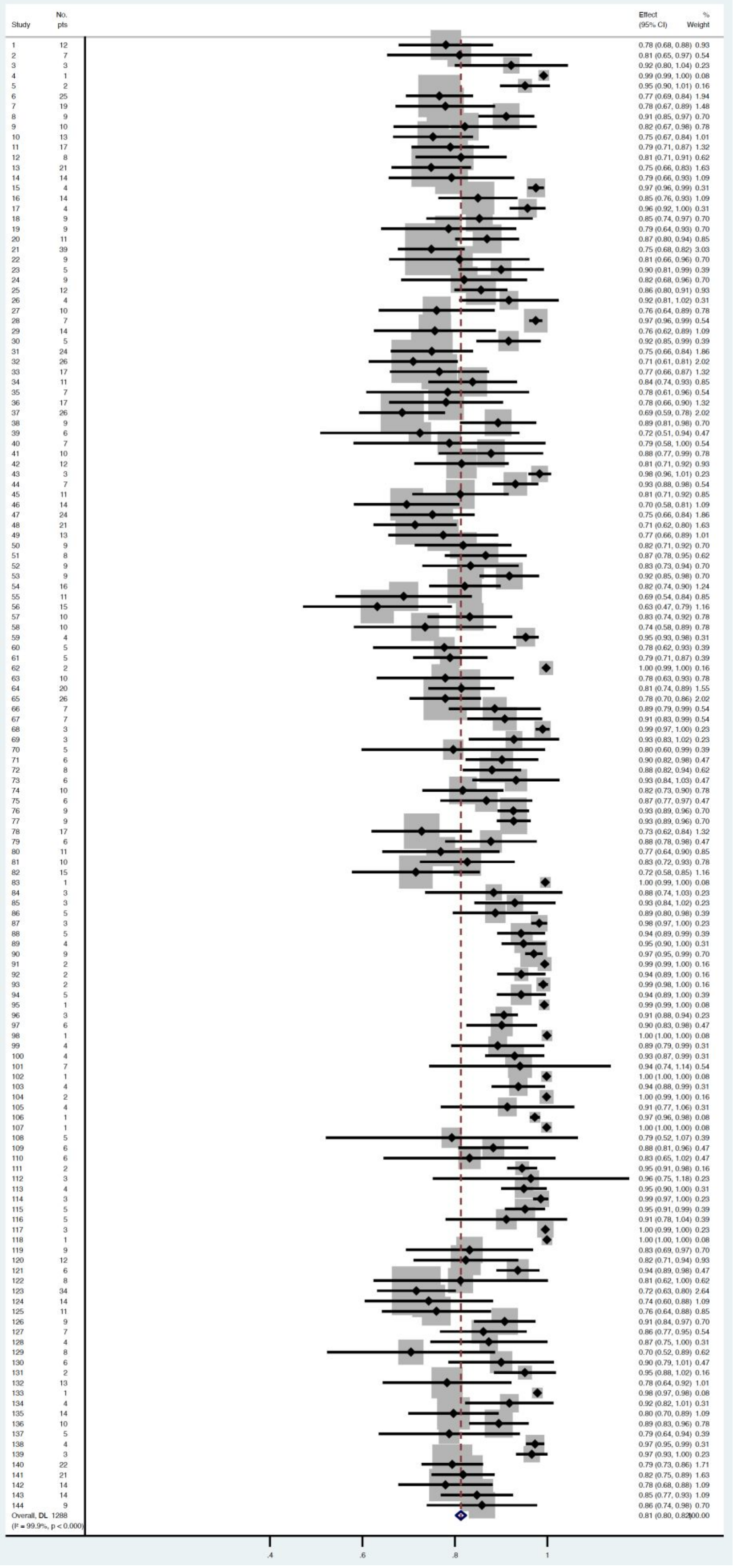

Tables

Table S1: Transparent reporting of a multivariable prediction model for individual prognosis or diagnosis (TRIPOD+AI) checklist for the reporting of prediction model studies

Section/topic	Item	Development/ Evaluation*	Checklist item
**Title**			
Title	1	D;E	Identify the study as developing or evaluating the performance of a multivariable prediction model, the target population, and the outcome to be predicted: Page 1
**Abstract**			
Abstract	2	D;E	See TRIPOD+AI for Abstracts checklist; Complete
**Introduction**			
Background	3a	D;E	Explain the healthcare context (including whether diagnostic or prognostic) and rationale for developing or evaluating the prediction model, including references to existing models: Introduction; Page 6
	3b	D;E	Describe the target population and the intended purpose of the prediction model in the context of the care pathway, including its intended users (eg, healthcare professionals, patients, public): Introduction; Page 6
	3c	D;E	Describe any known health inequalities between sociodemographic groups: Introduction; Page 6
Objectives	4	D;E	Specify the study objectives, including whether the study describes the development or validation of a prediction model (or both); Introduction: Page 7
**Methods**			
Data	5a	D;E	Describe the sources of data separately for the development and evaluation datasets (eg, randomised trial, cohort, routine care or registry data), the rationale for using these data, and representativeness of the data; Methods; Page 8/9
	5b	D;E	Specify the dates of the collected participant data, including start and end of participant accrual; and, if applicable, end of follow-up; Methods; Page 9

Section/topic	Item	Development/ Evaluation*	Checklist item
Participants	6a	D;E	Specify key elements of the study setting (eg, primary care, secondary care, general population) including the number and location of centres; Methods; Page 8/9
	6b	D;E	Describe the eligibility criteria for study participants; Methods; Page 8
	6c	D;E	Give details of any treatments received, and how they were handled during model development or evaluation, if relevant; N/A
Data preparation	7	D;E	Describe any data pre-processing and quality checking, including whether this was similar across relevant sociodemographic groups; Methods; Page 8
Outcome	8a	D;E	Clearly define the outcome that is being predicted and the time horizon, including how and when assessed, the rationale for choosing this outcome, and whether the method of outcome assessment is consistent across sociodemographic groups: Methods; Page 9
	8b	D;E	If outcome assessment requires subjective interpretation, describe the qualifications and demographic characteristics of the outcome assessors; N/A
	8c	D;E	Report any actions to blind assessment of the outcome to be predicted; N/A
Predictors	9a	D	Describe the choice of initial predictors (eg, literature, previous models, all available predictors) and any pre-selection of predictors before model building; Methods; Page 9
	9b	D;E	Clearly define all predictors, including how and when they were measured (and any actions to blind assessment of predictors for the outcome and other predictors); Methods; Page 9 and Table One
	9c	D;E	If predictor measurement requires subjective interpretation, describe the qualifications and demographic characteristics of the predictor assessors; N/A
Sample size	10	D;E	Explain how the study size was arrived at (separately for development and evaluation), and justify that the study size was sufficient to answer

Section/topic	Item	Development/ Evaluation*	Checklist item
			the research question. Include details of any sample size calculation; Methods; Page 10
Missing data	11	D;E	Describe how missing data were handled. Provide reasons for omitting any data; Methods; Page 10
Analytical methods	12a	D	Describe how the data were used (eg, for development and evaluation of model performance) in the analysis, including whether the data were partitioned, considering any sample size requirements; Methods; Page 10/11
	12b	D	Depending on the type of model, describe how predictors were handled in the analyses (functional form, rescaling, transformation, or any standardisation): Methods; Page 10/11
	12c	D	Specify the type of model, rationale†, all model building steps, including any hyperparameter tuning, and method for internal validation; Methods; Page 10/11
	12d	D;E	Describe if and how any heterogeneity in estimates of model parameter values and model performance was handled and quantified across clusters (eg, hospitals, countries). See TRIPOD-Cluster for additional considerations‡; Methods; Page 11
	12e	D;E	Specify all measures and plots used (and their rationale) to evaluate model performance (eg, discrimination, calibration, clinical utility) and, if relevant, to compare multiple models; Methods; Page 10/11
	12f	E	Describe any model updating (eg, recalibration) arising from the model evaluation, either overall or for particular sociodemographic groups or settings; N/A
	12g	E	For model evaluation, describe how the model predictions were calculated (eg, formula, code, object, application programming interface): N/A
Class imbalance	13	D;E	If class imbalance methods were used, state why and how this was done, and any subsequent methods to recalibrate the model or the model predictions; N/A
Fairness	14	D;E	Describe any approaches that were used to address model fairness and their rationale; N/A

Section/topic	Item	Development/ Evaluation*	Checklist item
Model output	15	D	Specify the output of the prediction model (eg, probabilities, classification). Provide details and rationale for any classification and how the thresholds were identified; Results page 12: Tables 4 and 6
Training versus evaluation	16	D;E	Identify any differences between the development and evaluation data in healthcare setting, eligibility criteria, outcome, and predictors; Nil
Ethical approval	17	D;E	Name the institutional research board or ethics committee that approved the study and describe the participant informed consent or the ethics committee waiver of informed consent; Declarations: Page 3
**Open science**			
Funding	18a	D;E	Give the source of funding and the role of the funders for the present study: Declarations; Page 2
Conflicts of interest	18b	D;E	Declare any conflicts of interest and financial disclosures for all authors: Declarations; Page 2
Protocol	18c	D;E	Indicate where the study protocol can be accessed or state that a protocol was not prepared; Methods: Page 8
Registration	18d	D;E	Provide registration information for the study, including register name and registration number, or state that the study was not registered; Nil
Data sharing	18e	D;E	Provide details of the availability of the study data: Declarations' Page 3
Code sharing	18f	D;E	Provide details of the availability of the analytical code§; Declarations: Page 3
**Patient and public involvement**			
Patient and public involvement	19	D;E	Provide details of any patient and public involvement during the design, conduct, reporting, interpretation, or dissemination of the study or state no involvement; Methods; Page 8 and throughout
**Result**			
Participants	20a	D;E	Describe the flow of participants through the study, including the number of participants with and without the outcome and, if applicable, a

Section/topic	Item	Development/ Evaluation*	Checklist item
			summary of the follow-up time. A diagram may be helpful; Results: Page 12
	20b	D;E	Report the characteristics overall and, where applicable, for each data source or setting, including the key dates, key predictors (including demographics), treatments received, sample size, number of outcome events, follow-up time, and amount of missing data. A table may be helpful. Report any differences across key demographic groups; Table 3
	20c	E	For model evaluation, show a comparison with the development data of the distribution of important predictors (demographics, predictors, and outcome); N/A
Model development	21	D;E	Specify the number of participants and outcome events in each analysis (eg, for model development, hyperparameter tuning, model evaluation); Table 3 and Table 4
Model specification	22	D	Provide details of the full prediction model (eg, formula, code, object, application programming interface) to allow predictions in new individuals and to enable third party evaluation and implementation, including any restrictions to access or reuse (eg, freely available, proprietary); Table 4
Model performance	23a	D;E	Report model performance estimates with confidence intervals, including for any key subgroups (eg, sociodemographic). Consider plots to aid presentation; Results Page 13; Table 5; OSM Figures S2/S3
	23b	D;E	If examined, report results of any heterogeneity in model performance across clusters. See TRIPOD-Cluster for additional details‡ Results; Page 13; OSM Figures S2/S3
Model updating	24	E	Report the results from any model updating, including the updated model and subsequent performance; N/A
**Discussion**			
Interpretation	25	D;E	Give an overall interpretation of the main results, including issues of fairness in the context of the

Section/topic	Item	Development/ Evaluation*	Checklist item
			objectives and previous studies; Discussion; Page 15
Limitations	26	D;E	Discuss any limitations of the study (such as a non-representative sample, sample size, overfitting, missing data) and their effects on any biases, statistical uncertainty, and generalisability; Discussion; Page 16
Usability of the model in the context of current care	27a	D	Describe how poor quality or unavailable input data (eg, predictor values) should be assessed and handled when implementing the prediction model' Discussion; Page 16
	27b	D	Specify whether users will be required to interact in the handling of the input data or use of the model, and what level of expertise is required of users; Discussion; Page 16/17
	27c	D;E	Discuss any next steps for future research, with a specific view to applicability and generalisability of the model; Discussion; Page 16/17

TRIPOD=Transparent Reporting of a multivariable prediction model for Individual Prognosis Or Diagnosis; AI=artificial intelligence.

- * D=items relevant only to the development of a prediction model; E=items relating solely to the evaluation of a prediction model; D;E=items applicable to both the development and evaluation of a prediction model.
- † Separately for all model building approaches.
- ‡ TRIPOD-Cluster is a checklist of reporting recommendations for studies developing or validating models that explicitly account for clustering or explore heterogeneity in model performance (eg, at different hospitals or centres).
- § Relates to the analysis code, for example, any data cleaning, feature engineering, model building, and evaluation.
- ¶ Relates to the code to implement the model to get estimates of risk for a new individual.

Table S2: International Classification of Diseases, Tenth Revision (ICD-10) codes used to define drug-related deaths

Description	ICD-10 Codes
Mental and behavioural disorders due to drug use (excluding alcohol and tobacco)	F11–F16, F18–F19
Accidental poisoning by drugs, medicaments and biological substances	X40–X44
Intentional self-poisoning by drugs, medicaments and biological substances	X60–X64
Assault by drugs, medicaments and biological substances	X85
Poisoning by drugs, medicaments and biological substances, undetermined intent	Y10–Y14

Table S3: Adjusted hazard ratios (95% confidence interval) for the complete case analysis of six month all-cause and drug-related mortality in individuals with opioid use disorder presenting to community addiction services in England

		**All-cause mortality**	**Drug-related mortality**
Age[1]		1.00 (1.00 - 1.00)	N/A
Sex	Female	Reference	Reference
	Male	1.07 (0.87 - 1.31)	0.93 (0.72 - 1.21)
History of injecting behavior	Never injected	Reference	Reference
	Previously injected (but not currently)	1.81 (1.43 - 2.29)	2.08 (1.50 - 2.88)
	Currently injecting	1.56 (1.22 - 2.00)	1.83 (1.30 - 2.57)
Hepatitis C RNA Positivity	Negative	Reference	Reference
	Positive	2.46 (2.02 - 3.00)	2.29 (1.76 - 2.99)
Problematic alcohol use	No	Reference	Reference
	Yes	1.43 (1.14 - 1.78)	1.31 (0.96 - 1.79)
Problematic benzodiazepine use	No	Reference	Reference
	Yes	1.26 (0.94 - 1.69)	1.70 (1.20 - 2.40)
Accommodation need	No housing problem	Reference	Reference
	Housing problem	1.29 (1.04 - 1.61)	1.32 (0.99 - 1.76)
	Urgent housing problem - NFA	1.21 (0.95 - 1.54)	1.19 (0.87 - 1.64)
Prison referral	Not referred from prison	Reference	Reference
	Referred from prison	1.59 (1.28 - 1.97)	1.91 (1.46 - 2.50)
Acute inpatient hospital admission	None	Reference	Reference
	Previous admission	1.06 (0.87 - 1.29)	0.95 (0.74 - 1.22)
Mental health inpatient hospital admission	None	Reference	Reference
	Previous admission	3.48 (1.44 - 8.42)	5.54 (2.05 - 14.92)

Previous history of addiction treatment	First treatment episode	Reference	Reference
	Previous treatment episode	1.92 (1.52 - 2.43)	1.93 (1.41 - 2.64)

1 Modelled as a cubic function in all-cause mortality model

S4. Proportion of missing values imputed as variable values following Multiple Imputation by Chained Equations (MICE)

		**Proportion of iterations imputed (%)**		
		**Full sample**	**Died due to any cause within six months**	**Died due to a drug-related cause within six months**
History of injecting behavior	Total missing (n)	1,895	26	11
	Never injected	42.6	42.4	45.4
	Previously injected (but not currently)	30.7	30.7	27.3
	Currently injecting	26.7	26.9	27.3
HIV Positivity	Total missing (n)	136,020	2,145	1,141
	Negative	97.9	97.6	97.8
	Positive	2.1	2.4	2.2
Hepatitis C RNA Positivity	Missing	138,690	1,862	977
	Negative	90.7	87.8	87.5
	Positive	9.3	12.2	12.5
Accommodation Need	Total missing (n)	25,671	64	22
	No housing problem	72.2	68.8	59.1
	Housing problem	15.3	18.1	27.3
	Urgent housing problem - NFA	12.5	13.1	13.6
Prison referral	Total missing (n)	1,278	17	9
	Not referred from prison	86.4	87.4	85.9
	Referred from prison	13.6	12.6	14.1

References

1. Collins GS, Moons KGM, Dhiman P, Riley RD, Beam AL, Van Calster B, et al. TRIPOD+AI statement: updated guidance for reporting clinical prediction models that use regression or machine learning methods. BMJ. 2024;385:e078378.

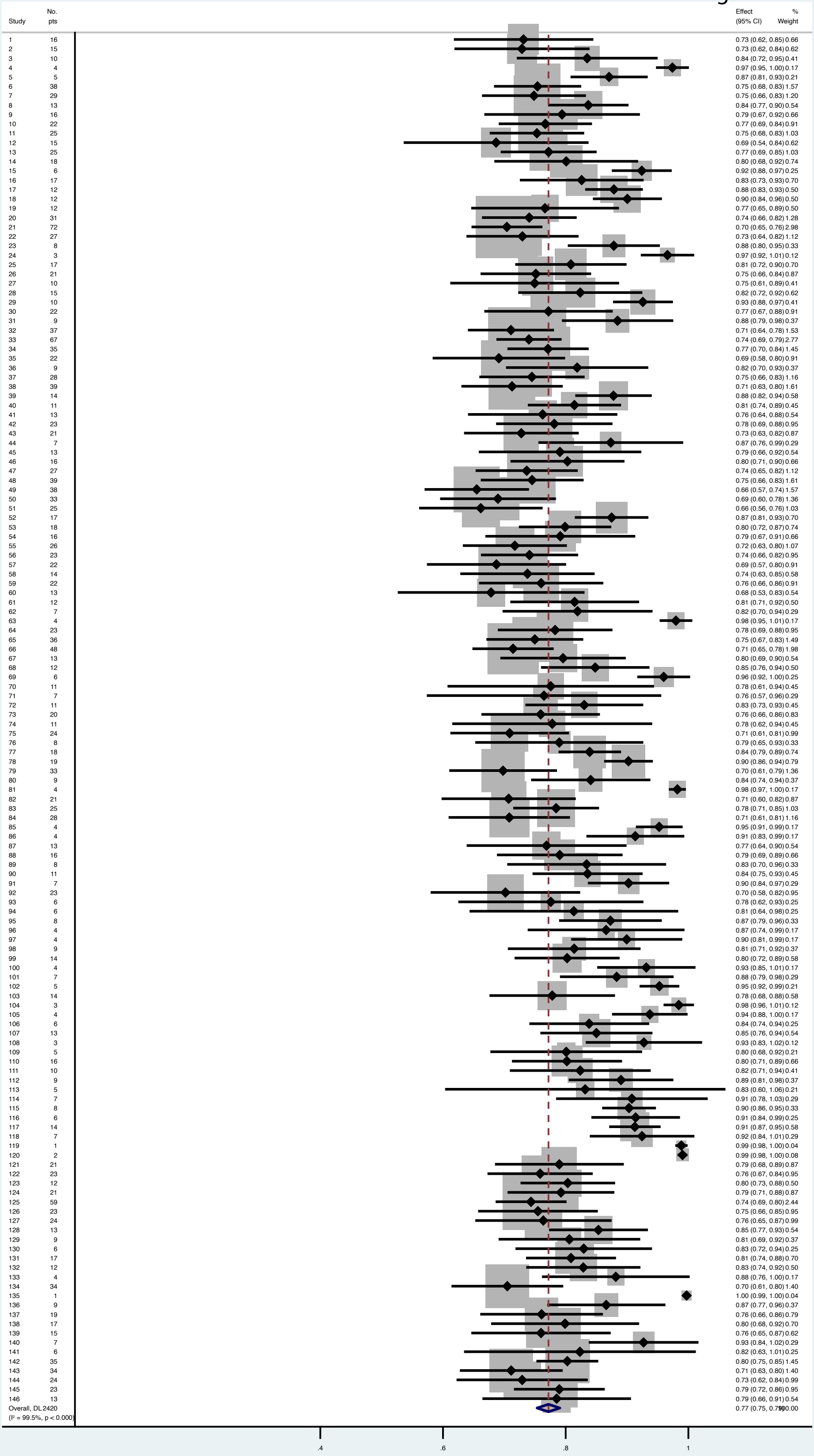
Study
No. pts
Effect (95% CI)
% Weight
1 16 0.73 (0.62, 0.85) 0.66
2 15 0.73 (0.62, 0.84) 0.62
3 10 0.84 (0.72, 0.95) 0.41
4 4 0.97 (0.95, 1.00) 0.17
5 5 0.87 (0.81, 0.93) 0.21
6 38 0.75 (0.68, 0.83) 1.57
7 29 0.75 (0.66, 0.83) 1.20
8 13 0.84 (0.77, 0.90) 0.54
9 16 0.79 (0.67, 0.92) 0.66
10 22 0.77 (0.69, 0.84) 0.91
11 25 0.75 (0.68, 0.83) 1.03
12 15 0.69 (0.54, 0.84) 0.62
13 25 0.77 (0.69, 0.85) 1.03
14 18 0.80 (0.68, 0.92) 0.74
15 6 0.92 (0.88, 0.97) 0.25
16 17 0.83 (0.73, 0.93) 0.70
17 12 0.88 (0.83, 0.93) 0.50
18 12 0.90 (0.84, 0.96) 0.50
19 12 0.77 (0.65, 0.89) 0.50
20 31 0.74 (0.66, 0.82) 1.28
21 72 0.70 (0.65, 0.76) 2.98
22 27 0.73 (0.64, 0.82) 1.12
23 8 0.88 (0.80, 0.95) 0.33
24 3 0.97 (0.92, 1.01) 0.12
25 17 0.81 (0.72, 0.90) 0.70
26 21 0.75 (0.66, 0.84) 0.87
27 10 0.75 (0.61, 0.89) 0.41
28 15 0.82 (0.72, 0.92) 0.62
29 10 0.93 (0.88, 0.97) 0.41
30 22 0.77 (0.67, 0.88) 0.91
31 9 0.88 (0.79, 0.98) 0.37
32 37 0.71 (0.64, 0.78) 1.53
33 67 0.74 (0.69, 0.79) 2.77
34 35 0.77 (0.70, 0.84) 1.45
35 22 0.69 (0.58, 0.80) 0.91
36 9 0.82 (0.70, 0.93) 0.37
37 28 0.75 (0.66, 0.83) 1.16
38 39 0.71 (0.63, 0.80) 1.61
39 14 0.88 (0.82, 0.94) 0.58
40 11 0.81 (0.74, 0.89) 0.45
41 13 0.76 (0.64, 0.88) 0.54
42 23 0.78 (0.69, 0.88) 0.95
43 21 0.73 (0.63, 0.82) 0.87
44 7 0.87 (0.76, 0.99) 0.29
45 13 0.79 (0.66, 0.92) 0.54
46 16 0.80 (0.71, 0.90) 0.66
47 27 0.74 (0.65, 0.82) 1.12
48 39 0.75 (0.66, 0.83) 1.61
49 38 0.66 (0.57, 0.74) 1.57
50 33 0.69 (0.60, 0.78) 1.36
51 25 0.66 (0.56, 0.76) 1.03
52 17 0.87 (0.81, 0.93) 0.70
53 18 0.80 (0.72, 0.87) 0.74
54 16 0.79 (0.67, 0.91) 0.66
55 26 0.72 (0.63, 0.80) 1.07
56 23 0.74 (0.66, 0.82) 0.95
57 22 0.69 (0.57, 0.80) 0.91
58 14 0.74 (0.63, 0.85) 0.58
59 22 0.76 (0.66, 0.86) 0.91
60 13 0.68 (0.53, 0.83) 0.54
61 12 0.81 (0.71, 0.92) 0.50
62 7 0.82 (0.70, 0.94) 0.29
63 4 0.98 (0.95, 1.01) 0.17
64 23 0.78 (0.69, 0.88) 0.95
65 36 0.75 (0.67, 0.83) 1.49
66 48 0.71 (0.65, 0.78) 1.98
67 13 0.80 (0.69, 0.90) 0.54
68 12 0.85 (0.76, 0.94) 0.50
69 6 0.96 (0.92, 1.00) 0.25
70 11 0.78 (0.61, 0.94) 0.45
71 7 0.76 (0.57, 0.96) 0.29
72 11 0.83 (0.73, 0.93) 0.45
73 20 0.76 (0.66, 0.86) 0.83
74 11 0.78 (0.62, 0.94) 0.45
75 24 0.71 (0.61, 0.81) 0.99
76 8 0.79 (0.65, 0.93) 0.33
77 18 0.84 (0.79, 0.89) 0.74
78 19 0.90 (0.86, 0.94) 0.79
79 33 0.70 (0.61, 0.79) 1.36
80 9 0.84 (0.74, 0.94) 0.37
81 4 0.98 (0.97, 1.00) 0.17
82 21 0.71 (0.60, 0.82) 0.87
83 25 0.78 (0.71, 0.85) 1.03
84 28 0.71 (0.61, 0.81) 1.16
85 4 0.95 (0.91, 0.99) 0.17
86 4 0.91 (0.83, 0.99) 0.17
87 13 0.77 (0.64, 0.90) 0.54
88 16 0.79 (0.69, 0.89) 0.66
89 8 0.83 (0.70, 0.96) 0.33
90 11 0.84 (0.75, 0.93) 0.45
91 7 0.90 (0.84, 0.97) 0.29
92 23 0.70 (0.58, 0.82) 0.95
93 6 0.78 (0.62, 0.93) 0.25
94 6 0.81 (0.64, 0.98) 0.25
95 8 0.87 (0.79, 0.96) 0.33
96 4 0.87 (0.74, 0.99) 0.17
97 4 0.90 (0.81, 0.99) 0.17
98 9 0.81 (0.71, 0.92) 0.37
99 14 0.80 (0.72, 0.89) 0.58
100 4 0.93 (0.85, 1.01) 0.17
101 7 0.88 (0.79, 0.98) 0.29
102 5 0.95 (0.92, 0.99) 0.21
103 14 0.78 (0.68, 0.88) 0.58
104 3 0.98 (0.96, 1.01) 0.12
105 4 0.94 (0.88, 1.00) 0.17
106 6 0.84 (0.74, 0.94) 0.25
107 13 0.85 (0.76, 0.94) 0.54
108 3 0.93 (0.83, 1.02) 0.12
109 5 0.80 (0.68, 0.92) 0.21
110 16 0.80 (0.71, 0.89) 0.66
111 10 0.82 (0.71, 0.94) 0.41
112 9 0.89 (0.81, 0.98) 0.37
113 5 0.83 (0.60, 1.06) 0.21
114 7 0.91 (0.78, 1.03) 0.29
115 8 0.90 (0.86, 0.95) 0.33
116 6 0.91 (0.84, 0.99) 0.25
117 14 0.91 (0.87, 0.95) 0.58
118 7 0.92 (0.84, 1.01) 0.29
119 1 0.99 (0.98, 1.00) 0.04
120 2 0.99 (0.98, 1.00) 0.08
121 21 0.79 (0.68, 0.89) 0.87
122 23 0.76 (0.67, 0.84) 0.95
123 12 0.80 (0.73, 0.88) 0.50
124 21 0.79 (0.71, 0.88) 0.87
125 59 0.74 (0.69, 0.80) 2.44
126 23 0.75 (0.66, 0.85) 0.95
127 24 0.76 (0.65, 0.87) 0.99
128 13 0.85 (0.77, 0.93) 0.54
129 9 0.81 (0.69, 0.92) 0.37
130 6 0.83 (0.72, 0.94) 0.25
131 17 0.81 (0.74, 0.88) 0.70
132 12 0.83 (0.74, 0.92) 0.50
133 4 0.88 (0.76, 1.00) 0.17
134 34 0.70 (0.61, 0.80) 1.40
135 1 1.00 (0.99, 1.00) 0.04
136 9 0.87 (0.77, 0.96) 0.37
137 19 0.76 (0.66, 0.86) 0.79
138 17 0.80 (0.68, 0.92) 0.70
139 15 0.76 (0.65, 0.87) 0.62
140 7 0.93 (0.84, 1.02) 0.29
141 6 0.82 (0.63, 1.01) 0.25
142 35 0.80 (0.75, 0.85) 1.45
143 34 0.71 (0.63, 0.80) 1.40
144 24 0.73 (0.62, 0.84) 0.99
145 23 0.79 (0.72, 0.86) 0.95
146 13 0.79 (0.66, 0.91) 0.54
Overall, DL 2420 0.77 (0.75, 0.79) 100.00
(I² = 99.5%, p < 0.000)
.4
.6
.8
1

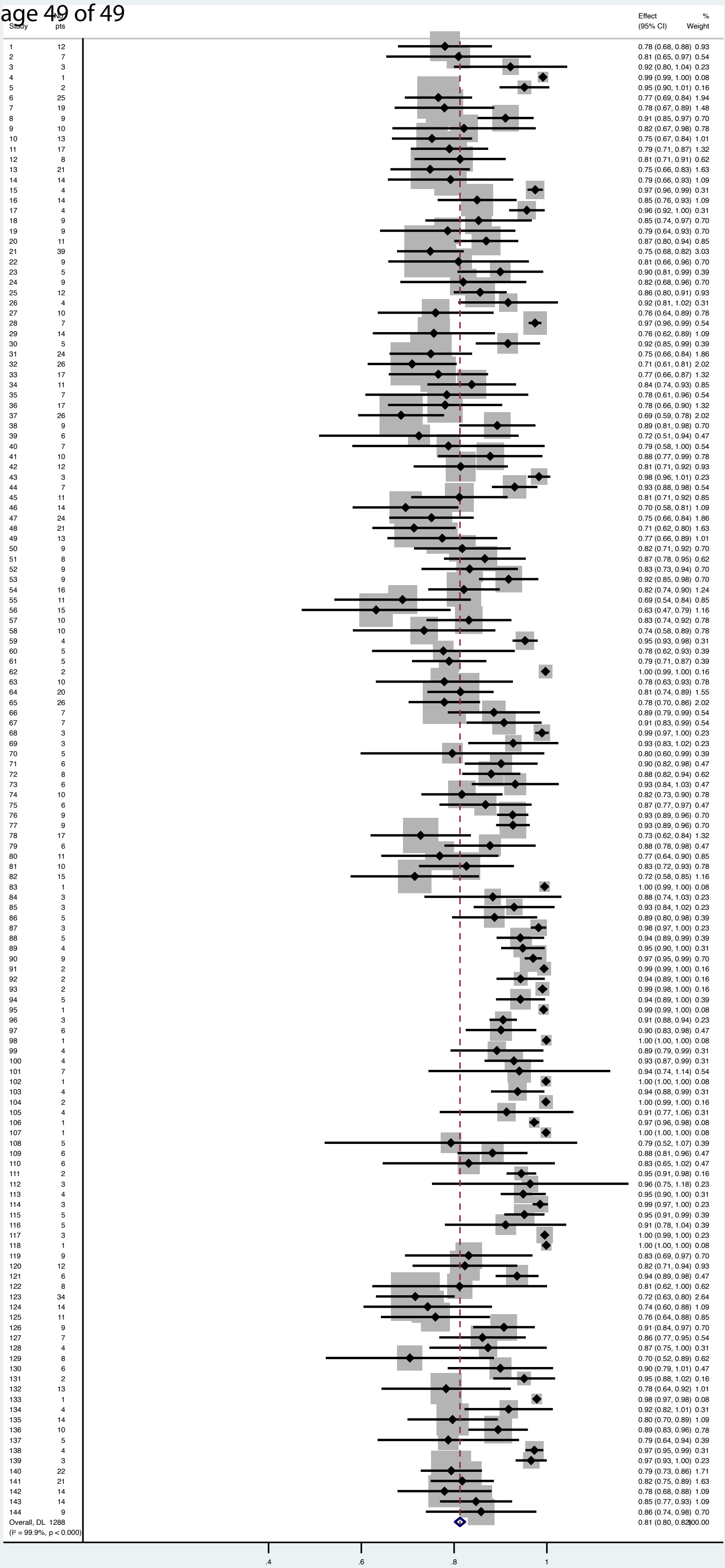
Study
No pts
Effect (95% CI)
% Weight
1 12 0.78 (0.68, 0.88) 0.93
2 7 0.81 (0.65, 0.97) 0.54
3 3 0.92 (0.80, 1.04) 0.23
4 1 0.99 (0.99, 1.00) 0.08
5 2 0.95 (0.90, 1.01) 0.16
6 25 0.77 (0.69, 0.84) 1.94
7 19 0.78 (0.67, 0.89) 1.48
8 9 0.91 (0.85, 0.97) 0.70
9 10 0.82 (0.67, 0.98) 0.78
10 13 0.75 (0.67, 0.84) 1.01
11 17 0.79 (0.71, 0.87) 1.32
12 8 0.81 (0.71, 0.91) 0.62
13 21 0.75 (0.66, 0.83) 1.63
14 14 0.79 (0.66, 0.93) 1.09
15 4 0.97 (0.96, 0.99) 0.31
16 14 0.85 (0.76, 0.93) 1.09
17 4 0.96 (0.92, 1.00) 0.31
18 9 0.85 (0.74, 0.97) 0.70
19 9 0.79 (0.64, 0.93) 0.70
20 11 0.87 (0.80, 0.94) 0.85
21 39 0.75 (0.68, 0.82) 3.03
22 9 0.81 (0.66, 0.96) 0.70
23 5 0.90 (0.81, 0.99) 0.39
24 9 0.82 (0.68, 0.96) 0.70
25 12 0.86 (0.80, 0.91) 0.93
26 4 0.92 (0.81, 1.02) 0.31
27 10 0.76 (0.64, 0.89) 0.78
28 7 0.97 (0.96, 0.99) 0.54
29 14 0.76 (0.62, 0.89) 1.09
30 5 0.92 (0.85, 0.99) 0.39
31 24 0.75 (0.66, 0.84) 1.86
32 26 0.71 (0.61, 0.81) 2.02
33 17 0.77 (0.66, 0.87) 1.32
34 11 0.84 (0.74, 0.93) 0.85
35 7 0.78 (0.61, 0.96) 0.54
36 17 0.78 (0.66, 0.90) 1.32
37 26 0.69 (0.59, 0.78) 2.02
38 9 0.89 (0.81, 0.98) 0.70
39 6 0.72 (0.51, 0.94) 0.47
40 7 0.79 (0.58, 1.00) 0.54
41 10 0.88 (0.77, 0.99) 0.78
42 12 0.81 (0.71, 0.92) 0.93
43 3 0.98 (0.96, 1.01) 0.23
44 7 0.93 (0.88, 0.98) 0.54
45 11 0.81 (0.71, 0.92) 0.85
46 14 0.70 (0.58, 0.81) 1.09
47 24 0.75 (0.66, 0.84) 1.86
48 21 0.71 (0.62, 0.80) 1.63
49 13 0.77 (0.66, 0.89) 1.01
50 9 0.82 (0.71, 0.92) 0.70
51 8 0.87 (0.78, 0.95) 0.62
52 9 0.83 (0.73, 0.94) 0.70
53 9 0.92 (0.85, 0.98) 0.70
54 16 0.82 (0.74, 0.90) 1.24
55 11 0.69 (0.54, 0.84) 0.85
56 15 0.63 (0.47, 0.79) 1.16
57 10 0.83 (0.74, 0.92) 0.78
58 10 0.74 (0.58, 0.89) 0.78
59 4 0.95 (0.93, 0.98) 0.31
60 5 0.78 (0.62, 0.93) 0.39
61 5 0.79 (0.71, 0.87) 0.39
62 2 1.00 (0.99, 1.00) 0.16
63 10 0.78 (0.63, 0.93) 0.78
64 20 0.81 (0.74, 0.89) 1.55
65 26 0.78 (0.70, 0.86) 2.02
66 7 0.89 (0.79, 0.99) 0.54
67 7 0.91 (0.83, 0.99) 0.54
68 3 0.99 (0.97, 1.00) 0.23
69 3 0.93 (0.83, 1.02) 0.23
70 5 0.80 (0.60, 0.99) 0.39
71 6 0.90 (0.82, 0.98) 0.47
72 8 0.88 (0.82, 0.94) 0.62
73 6 0.93 (0.84, 1.03) 0.47
74 10 0.82 (0.73, 0.90) 0.78
75 6 0.87 (0.77, 0.97) 0.47
76 9 0.93 (0.89, 0.96) 0.70
77 9 0.93 (0.89, 0.96) 0.70
78 17 0.73 (0.62, 0.84) 1.32
79 6 0.88 (0.78, 0.98) 0.47
80 11 0.77 (0.64, 0.90) 0.85
81 10 0.83 (0.72, 0.93) 0.78
82 15 0.72 (0.58, 0.85) 1.16
83 1 1.00 (0.99, 1.00) 0.08
84 3 0.88 (0.74, 1.03) 0.23
85 3 0.93 (0.84, 1.02) 0.23
86 5 0.89 (0.80, 0.98) 0.39
87 3 0.98 (0.97, 1.00) 0.23
88 5 0.94 (0.89, 0.99) 0.39
89 4 0.95 (0.90, 1.00) 0.31
90 9 0.97 (0.95, 0.99) 0.70
91 2 0.99 (0.99, 1.00) 0.16
92 2 0.94 (0.89, 1.00) 0.16
93 2 0.99 (0.98, 1.00) 0.16
94 5 0.94 (0.89, 1.00) 0.39
95 1 0.99 (0.99, 1.00) 0.08
96 3 0.91 (0.88, 0.94) 0.23
97 6 0.90 (0.83, 0.98) 0.47
98 1 1.00 (1.00, 1.00) 0.08
99 4 0.89 (0.79, 0.99) 0.31
100 4 0.93 (0.87, 0.99) 0.31
101 7 0.94 (0.74, 1.14) 0.54
102 1 1.00 (1.00, 1.00) 0.08
103 4 0.94 (0.88, 0.99) 0.31
104 2 1.00 (0.99, 1.00) 0.16
105 4 0.91 (0.77, 1.06) 0.31
106 1 0.97 (0.96, 0.98) 0.08
107 1 1.00 (1.00, 1.00) 0.08
108 5 0.79 (0.52, 1.07) 0.39
109 6 0.88 (0.81, 0.96) 0.47
110 6 0.83 (0.65, 1.02) 0.47
111 2 0.95 (0.91, 0.98) 0.16
112 3 0.96 (0.75, 1.18) 0.23
113 4 0.95 (0.90, 1.00) 0.31
114 3 0.99 (0.97, 1.00) 0.23
115 5 0.95 (0.91, 0.99) 0.39
116 5 0.91 (0.78, 1.04) 0.39
117 3 1.00 (0.99, 1.00) 0.23
118 1 1.00 (1.00, 1.00) 0.08
119 9 0.83 (0.69, 0.97) 0.70
120 12 0.82 (0.71, 0.94) 0.93
121 6 0.94 (0.89, 0.98) 0.47
122 8 0.81 (0.62, 1.00) 0.62
123 34 0.72 (0.63, 0.80) 2.64
124 14 0.74 (0.60, 0.88) 1.09
125 11 0.76 (0.64, 0.88) 0.85
126 9 0.91 (0.84, 0.97) 0.70
127 7 0.86 (0.77, 0.95) 0.54
128 4 0.87 (0.75, 1.00) 0.31
129 8 0.70 (0.52, 0.89) 0.62
130 6 0.90 (0.79, 1.01) 0.47
131 2 0.95 (0.88, 1.02) 0.16
132 13 0.78 (0.64, 0.92) 1.01
133 1 0.98 (0.97, 0.98) 0.08
134 4 0.92 (0.82, 1.01) 0.31
135 14 0.80 (0.70, 0.89) 1.09
136 10 0.89 (0.83, 0.96) 0.78
137 5 0.79 (0.64, 0.94) 0.39
138 4 0.97 (0.95, 0.99) 0.31
139 3 0.97 (0.93, 1.00) 0.23
140 22 0.79 (0.73, 0.86) 1.71
141 21 0.82 (0.75, 0.89) 1.63
142 14 0.78 (0.68, 0.88) 1.09
143 14 0.85 (0.77, 0.93) 1.09
144 9 0.86 (0.74, 0.98) 0.70
Overall, DL 1288 0.81 (0.80, 0.82) 100.00
(I² = 99.9%, p < 0.000)
.4
.6
.8
1
